# The undergraduate premedical experience in the United States: a critical review

**DOI:** 10.5116/ijme.5103.a8d3

**Published:** 2013-02-10

**Authors:** Katherine Y. Lin, Sonali Parnami, Andrea Fuhrel-Forbis, Renee R. Anspach, Brett Crawford, Raymond G. De Vries

**Affiliations:** 1 Department of Sociology, University of Michigan, Ann Arbor, MI, USA; 2 Center for Bioethics and Social Sciences in Medicine, University of Michigan, Ann Arbor, MI, USA; 3 School of Business Administration, Wayne State University, Detroit, MI, USA

**Keywords:** Premedical education, premedical syndrome, attrition from premedical track, hidden curriculum, professionalization, USA

## Abstract

**Objectives:**

To better understand the consequences of the premedical years for the character of (future) physicians by critically reviewing the empirical research done on the undergraduate premedical experience in the United States.

**Methods:**

We searched ERIC, JSTOR, PubMed, Scopus, ISI Web of Science, and PsycINFO from the earliest available date for empirical, peer-reviewed studies of premedical students in the United States. We then used qualitative methods to uncover overall themes present in this literature.

**Results:**

The initial literature search identified 1,168 articles, 19 of which were included for review. Reviewed articles were published between 1976 and 2010 with the majority published prior to 1990. Articles covered two broad topics: explaining attrition from the premedical track, and investigating the personality traits and stereotypes of premedical students. Self-selection bias and high attrition rates were among the limitations of the reviewed articles.

**Conclusions:**

There is very little current research on the premedical experience. Given the importance of the pre-medical years on the process of becoming a medical professional, it is imperative that we do more and better research on how the premedical experience shapes future physicians.

## Introduction

Scholars and policy makers have long been concerned with the selection and training of the future physician workforce--concerned not only with the academic competence of future doctors, but also with the quality of their character, including their ability to reason morally, to listen to patients, and to empathize with patients as whole persons, rather than seeing them as collections of genes, cells, and organs. While prior research in this area has largely considered the influences of the criteria for admission into medical school or how students are influenced by their medical training,^1^ more recently, scholars have begun to consider how the premedical years shape future physicians.^2^^-^^4^ One indicator of this new interest in the premedical years is the recent decision by the Association of American Medical Colleges to revise the Medical College Admissions Test (MCAT) to include questions on the social and behavioral sciences so as to encourage premedical students to study these topics.^5^

Empirical evidence has demonstrated that what happens to students prior to entering medical school affects their performance during medical school and beyond. For instance, there are documented correlations between premedical academic performance and pre-clinical academic performance in the UK,^6^^,^^7^ the US,^8^ and elsewhere.^9^ Others have argued that students enter medical school with already formed values and ethical points of view that may be difficult to influence or alter with current bioethics curricula in medical schools.^10^^,^^11^ Recent studies on physician depression and burnout also indicate that physician well-being is diminished by the stress of medical and premedical education.^12^^-^^14^ These studies and policy changes suggest that the premedical years have a significant influence on the character and well-being of physicians.

Most prior research on the premedical years has focused on how the academic experience – in particular, the performance in, and choice of, coursework during these years – influences outcomes during medical school years and beyond.^15^While important, the academic experience is only one aspect of the premedical years. Premedical education, like medical education, includes the formal curriculum, as well as informal and hidden curricula.^16^ The entire experience of the premedical years – including academic and formal curricular training as well as informal and co-curricular experiences, such as cooperating with and competing against classmates in required coursework, participating in a variety of extracurricular activities, and repeatedly examining their ambitions to become doctors – influences future physicians. Although there have been several reviews of how the academic performance of premedical students influences their academic performance in medical school and in practice,^17^^,^^18^ to our knowledge no similar synthesis of the literature on other aspects of the premedical experience has been done.

Our goal in this review is to critically synthesize what is known about the premedical experience in the United States in order to generate research questions for future scholarship.^19^ We chose to study premedical students in the United States because in the US the premedical years are well defined, offering a focused look at the experience of “being a premed”. What we learn here may be compared to other medical education systems where the “premedical years” occur in secondary school. In the US context, we reviewed studies of students in four-year post-secondary institutions. There are other avenues for gaining admission to American medical schools, but the vast majority of those who apply to US medical schools graduate from this type of institution.^20^

### Defining the premedical experience

Though there is no single or standard pathway into medical school in the United States, the simple act of planning to go to medical school subjects a student to a particular set of requirements and competitive pressures. The curricular requirements and extra-curricular expectations for gaining admission to medical school–established by individual medical schools and by the content areas of the MCAT – provide the overall structure of the premedical years. The premedical experience, however, encompasses all of the things students do inside and outside the classroom – strategizing, competing, and collaborating– to successfully master challenging academic material and satisfactorily meet requirements in hopes of putting together a successful medical school application. Thus, the premedical experience is determined by far more than one’s academic skill or personality - it is the informal, yet patterned and collective response to the requirements of securing a career as a physician.

In the United States, for instance, students must not only score well on the MCAT, but they must also do well in large “weeder” courses (most notably, organic chemistry, a course that separates the “smart” from the “not-so-smart” students) while carefully crafting a résumé of desirable extra-curricular activities, and balancing academic, social, and personal lives. Students in countries where admission to medical school occurs directly after secondary education face similar requirements. For example, students in the United Kingdom must take the required number of A-level courses in a variety of subjects, do well on an Aptitude Test (e.g. the UKCAT: UK Clinical Aptitude Test), and enhance their attractiveness to medical schools with participation in extracurricular activities. These requirements amount to a type of cultural pressure that contributes to the socialization of future physicians. Thus, the character of the next generation of physicians is forged long before students walk through the doors of medical school: medical socialization begins with the negotiation of the premedical years.

Participants in the premedical experience include not only those who will become medical students and practicing physicians, but also those who initially express interest in medical studies but end up pursuing other careers. Premedical students who choose not to apply to medical school are also crucial participants in the premedical experience, and also help shape the competitive pressures and collective experiences of all premedical students. Thus, studies that examine the premedical years by asking only medical students (i.e., those who have successfully gained entry to medical school) miss an essential part of the premedical experience. Similarly, studies that use data from students in special pipeline programs (i.e. BA/MD programs) may also not capture the full extent of the premedical experience since these students are subject to a different set of environmental pressures. In fact, many of these programs were established initially to combat competitive pressures present in the modal premedical experience.^21^Students in pipeline programs may have stronger ties to medical school upon matriculation into these programs, increased academic and support for medical studies, and face different competitive pressures as many programs do not require these students to take the MCAT for admission.^21^ For this reason, to accurately capture the premedical experience, we review studies of premedical students, excluding studies of students in pipeline programs, during their premedical years.

## Methods

We began our review by searching through databases relevant to medical education and social science research: ERIC, JSTOR, , Scopus, ISI Web of Science, and PsycINFO. Using intentionally broad search terms, we included appropriate combinations of keywords and controlled vocabulary terms relating to premedical education (See Appendix I: Databases searched & search strategies). No limits were placed on date, language or article type during the retrieval process (done on June 16, 2010). To ensure we did not miss a study that met our criteria, we also searched the references of all full-review articles for relevant studies.

**Figure 1:**
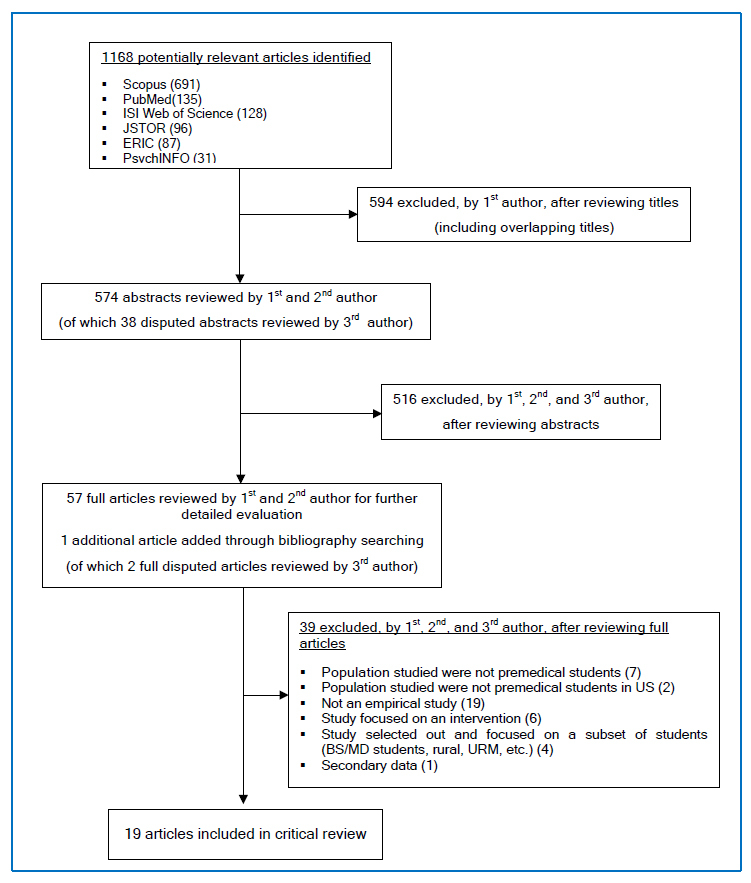
Selection of the articles for the critical review

The first author reviewed the 1,168 titles generated from the initial search and selected 574 relevant articles for abstract review (See Figure 1). Articles were discarded if the titles indicated the sample were not United States premedical students. The majority of the articles discarded used the term “premedical” or “undergraduate medical education” to refer to the first two years of formal medical training before clinical rotations rather than undergraduate education leading to a bachelor’s degree. Articles were included for abstract review if it was not clear from the title that the research question dealt with premedical students in the United States. The first and second authors reviewed abstracts from each of the articles to select articles for full text review; the third author was consulted to resolve disagreements. Persisting disagreements were resolved by discussion with all authors. The first and second authors then independently reviewed fifty-eight full-text articles to determine final inclusion (with the third author resolving disagreements and persisting disagreements resolved via group discussion). Nineteen articles met inclusion criteria for this review.

Our main selection criterion was that the study needed to have sampled premedical students during their premedical years (see Table 1). Given our focus on all premedical students (and not just those who succeeded in gaining admission to medical school) we excluded studies that sampled only medical students to draw conclusions about the premedical experience. Additionally, all articles examining premedical students in the US published in peer-reviewed journals were considered for inclusion, while monographs, book reviews, national reports, and conference proceedings were excluded. Because we are interested in learning about the premedical experience and not just premedical coursework, we excluded studies that did not gather primary data from premedical students – such as studies that examined only undergraduate students’ academic transcripts. We also excluded studies that exclusively examined students from combined BA/MD programs, post-baccalaureate programs, and special medical pipeline programs, since students in these programs already have guaranteed admission into medical school at the undergraduate level, or follow pathways into medical school that are unconventional, and therefore do not reflect the modal experience of premedical students.

The first author, assisted by the second author, summarized all articles and developed a typology of the overarching themes found in the literature. Themes were then grouped together into broader analytic questions that have been asked about the premedical experience. Since the study did not directly collect data from human subjects, research ethics approval was not obtained.

## Results

The nineteen articles we reviewed were published between 1976 and 2010 and varied greatly in focus and design (Table 2). The articles were methodologically diverse: twelve articles were based on large sample surveys of premedical students; five were based on qualitative interviews with smaller samples of premedical students, and two used a mixed-method approach. This empirical work on premedical students can be categorized into two broad research agendas: explaining attrition from the premedical track, and investigating the personality traits and stereotypes of premedical students.

**Table 1:** Inclusion and exclusion criteria for study

Inclusion criteria
§ Empirical, published study in a peer-reviewed journal
§ Focuses on premedical education in the United States
§ Study samples only “modal” premedical students
Notable exceptions include studies that include both a premedical sample AND another sample (premedical advisors, for example). If the results for premedical students were reported separately, this can be included.
§ Study collects primary data that reflects premedical student attitudes, perceptions, experiences, or proxy for these
Studies that use course enrolment data are not included, unless authors extrapolate something about the premedical experience from the course enrolment data. Descriptions of enrolment patterns in courses are not sufficient.
Exclusion criteria
§ Study is an evaluation or examination of a pipeline program or other “special programs,” such as those designed to boost ad-missions of a subset of premedical students into medical school, and only uses data from these types of students.
§ Study uses only samples of high school students, medical students, post-baccalaureate students, BA/MD students, students enrolled in special programs, or students who have al-ready been accepted to medical school
§ Written in a language other than English

### Attrition from the premedical track

Eleven of the nineteen articles reviewed attempted to explain why certain students entered their undergraduate studies with an interest in medicine but ended up pursuing other aspirations. These articles focused on why women and under-represented minorities disproportionately leave the premedical track. The earlier work in this group, done between the 1970s through the 1990s, found that from the beginning of their premedical years women were less certain than men about their medical career aspirations,^22^ or had less of a normative disincentive to abandon their original aspirations.^23^^,^^24^ Other work from the early 1990s found that premedical students, in general, decided not to apply to medical school either because they were no longer interested in medicine or because they realized that their previous expectations of what it would be like to be a physician no longer matched what they now viewed as the reality of a medical career.^25^^,^^26^ These studies, done more than 20 years ago, indicated that those who left the premedical track often did so as a result of a change in individual-level goals or aspirations.

**Table 2:** Studies included in critical review

Source	Research question	Method	Measures	Population	Sample size	Response rate	Methodological limitations
Barondess and Glaser 1993^26 ^	What are the attitudes that lead potential medical school applicants towards or away from medical school?	Survey / Cross-sectional	Attitudes towards medical careers--such importance of job satisfaction, stability of career, and ability to help those who are less fortunate in choosing a medical career	National sample of college students and premedical advisors from 100 US schools	Study 1: total sample 1780 students (1003 non-applicants to medical school; 500 medical school applicants; 227 qualified non-applicants); Study 2:93 premedical advisors	Study 1: Student groups: 84-89%; Study 2: Premedical advisors: 93%	Limited information provided regarding interview items or whether items were pretested.
Barr et al 2008^27^	What are the causes, among underrepresented minority students, of a decline in interest in pursuing a career in medicine?	Study 1: Survey/ Longitudinal; Study 2: Follow-up interviews with subset of Study 1 survey participants	Survey measure: 10-point scale of interest in premedical studies. Interviews asked about factors leading to a change in interest, the role of coursework in changing interests, and important skills or resources premedical students need to succeed	Incoming freshman college students who indicated interest in medicine and completed all 3 surveys, Stanford University	Study 1: 362 students; Study 2:68 students	Study 1: Initial response rate to survey invitation not reported; 34.3% of 1056 T1 respondents completed all 3 surveys; Study 2: Not reported	Limited generali-zability - sampled only from one university, which is among the most selective nationally; Students self-selected to take the survey; High rate of attrition over time
Barr et al 2010^28 ^	Do negative experiences in chemistry courses contribute to undergraduate students discontinuing premedical studies?	Study 1: Sur-vey/Repeated Measures; Study 2: Follow-up interviews with subset of survey participants	Survey measure: 10-point scale of interest in premedical studies. Interviews asked about factors leading to a change in interest, and to identify courses that discouraged interest	Incoming freshman college students who indicated an interest in medicine, UC Berkeley	Study 1: 1036 students; Study 2:63 students completed follow-up interviews	Study 1: Initial response rate to survey invitation not reported; 57% of the 1036 T1 respondents also responded at the end of their second year; Study 2: Not reported	Limited generali-zability- sampled only from one university, which is among the most selective nationally; Students self-selected to take the survey; Initial response rate not reported; High rate of attrition over time; Students not individually identified so unable to track individual change over time
Chuck 1996^32^	What are premedical students’ expectations from a medical career?	Survey/ Cross-sectional	Expectations of a medical career such as the ability of physicians to cure and help their patients, to have intellectually satisfying work, or to have to perform administrative duties	Study 1: premedical students, UC Berkeley and Cal State Hayward; Study 2; practicing physicians from similar geographic area	Study 1:84 premedical students; Study 2:251 practicing physicians	Study 1: premedical students: 100%; Study 2: physicians: 62%	Possible limited generalizability because this sample of premedical students may be more engaged than the general population of premedical students; No statistical tests of magnitude of between-subjects comparisons
Colquitt and Killian 1991^25^	Why do students who take the MCAT opt to not apply to medical school in the years 1986 and 1988?	Study 1: Secondary data analysis; Studies 2 & 3: Survey/ cross-sectional with open ended response	Reasons for not applying to medical school, attitudes about medical practice	Study 1: MCAT examinees; Studies 2 & 3: non-applicants to medical school (took MCAT but did not apply)	Study 1: secondary data analysis (n/a); Study 2: 539 non-applicants (1986); Study 3: 745 non-applicants (1988)	Study 1: secondary data analysis (n/a); Study 2: 35% (1986); Study 3: 32% (1988)	Self-selection bias possible due to low response rate (authors used weighted analysis to correct for non-response bias)
Conrad 1986^40^	How prevalent is the premedical cut-throat stereotype in reality? Despite a low prevalence, why does the myth still exist?	Interviews/ Cross-sectional	Asked interviewees about the premedical sub-culture, and conducted fieldwork in premedical settings	Premedical students, Brandeis University	30 premedical students	Potential participants approached– not reported	Half of sample was convenience sample; Limited generalizability to other institutions due to selective, private nature, small institutional size, and high focus on premedical program at Brandeis
Fiorentine 1987^23^	What causes the premedical persistence gap? Why do more men persist in premedical studies than women?	Study 1: Secondary data analysis; Study 2: Interviews/ Cross-sectional	Student academic achievement, persistence (continuing with premedical studies, application to medical school), interviews focus on why students want to become doctors	Study 1: academic transcripts of likely premedical students; Study 2: interviews with premedical students (GPA 2.50-3.50), SUNY Stony Brook	Study 1: total sample 673 students (323 females, 350 males); Study 2: total sample 27 students (16 females, 11 males)	Study 1: n/a; Study 2: Potential participants approached – not reported	“Premedical” as defined by author could include students who were not actually premedical; limited detail given of development of interview protocol or selection of interview participants; Study 2: Limited generalizability due to small sample size for interviews; Inferences and conclusions made from interview responses reach beyond scope of data
Fiorentine and Cole 1992^24^	Why do fewer women apply to medical school if they are as likely as men to get into and through medical school?	Study 1: Interviews/ Cross-sectional; Study 2: Interviews with subset from Study 1; Study 3: Interviews/ Cross-sectional with separate population	Student academic achievement (transcripts) and application to medical school status, self-reported measures of persistence, level of encouragement perceived, and family formation plans	Study 1: 542 persisting and defecting premedical students currently enrolled at time of study, SUNY Stony Brook; Study 2: 36 premedical students; Study 3: 62 high school students with premedical plans	Study 1: total sample 542 students (240 females, 302 males); Study 2: total sample 36 students (23 female, 13 male); Study 3: 62 students	Study 1: 542 of 543 (99.8%) students participated in telephone interview; Study 2: Potential participants approached – not reported; Study 3: 62 of 62 (100%) eligible interviews completed	Limited detail given of development of interview protocol or selection of interview participants; Selective attrition due to defection from premedical program; Lack of transparency in reporting results (study samples grouped)
Hackman et al 1979^33 ^	What are the perceptions of premedical students held by both premedical and non-premedical students?	Survey/ Cross-sectional with closed and open-ended responses	Ratings of students' feelings towards other student groups (i.e. Premed, pre-law, etc)	Undergraduate college students, Yale	Total sample 317 students (132 sophomores and 106 seniors randomly selected, 79 seniors who applied to medical school)	65.40%	Limited generalizability - sampled from one private selective university.
Horowitz 2010^39 ^	What achievement goal orientations are adopted by premedical students? How does this differ by subject, major, requirements?	Semi-structured interviews	Main interview question: "In which subject areas do you choose courses just to get an easy A and in which subject areas do you choose more challenging courses because of an interest or a desire to learn?"	Undergraduate college students who completed Organic chemistry during 2006-07 year at a small, all-male, liberal arts college contained within a private Jewish university in the northeastern U.S.	Total sample 30 students (87% premedical; 84% majoring in biology or chemistry.	31 (54.4%) of the 57 students completing organic chemistry agreed to participate – data from 30 interviews reported	Limited generalizability – low response rate, sampled from one small, all-male, private, northeastern US, Jewish university
Klink et al 2008^31 ^	Is there a relationship between premedical and non-premedical students’ perceived level of family support and confidence in their abilities to cope with premedical life?	Survey/ Cross-sectional	Scale of coping efficacy, self-reported measures of family support	Premedical, University of Wisconsin.	Total sample 238 students	57% of expected estimate of participants responded	Limited generalizability – low response rate, sampled from one large, competitive, Midwestern university; Study used limited dimensions of family support which may be related to coping efficacy.
Lewis 1985^38^	Does the career choice of medicine influence a premedical student to undertake certain types of coursework at a liberal arts college?	Study 1: Survey/ Cross-sectional; Study 2: Sur-vey/Longitudinal of subset from Study 1.	Self-reported measures of career goals, degree goals, college major, personality traits, and interactional styles	Study 1: Premedical and non-premedical students in three classes 1982, 1983, 1985, Macalester College, St. Olaf College; Study 2: Classes 1983 and 1985 resurveyed.	Study 1: Total sample 345 students (58 premedical, 99 biology/chem majors, 187 others); Study 2: Not reported.	Study 1 & Study 2: Potential participants approached – not reported	Study 1 & 2: Limited generalizability- sampled from two small, liberal arts colleges; Data presents limited means of testing study hypotheses; Study 2: No population data or report on loss to follow-up.
Lovecchio and Dundee 2002^29 ^	How do premedical and former premedical students differ on perceptions of a medical career and reasons for desiring a medical career? How does this differ by years in school and by gender?	Survey/Cross-sectional using two different questionnaires.	Self-reported career aspirations and reasons for leaving the premedical track	Current premedical students and former premedical students, McDaniel College	Total sample 97 students (44 former premedical, 53 current premedical)	100%	Limited generalizability because convenience sampling method, small sample size, and sampled from one, small, private liberal arts college; Upper-classmen disproportionately represented in both comparison groups.
Manaster et al 1976^35 ^	How do incoming premedical students’ attitudes about medicine compare to those who are currently applying to medical school? How does this differ by gender?	Survey/Cross-sectional	Self-reported measures of social background, perceptions of medicine and medical school, perception of family relations, parents, and self during childhood, personality items that assess anxiety, extraversion, and internality	Incoming premedical students and premedical students applying to medical school, University of Texas	Total sample 554 students (372 incoming premeds, 182 premeds applying to medical school)	Potential participants approached – not reported	Limited generalizability- sampled from one, large university; Skewed male/female ratio; Limited detail if/how questionnaire was pretested; How measured variables related to impact of collegiate experience on occupational goals not clearly defined.
McCranie and Lewis 1987^36^	What is the prevalence of Type A behavior among premedical students and students pursuing other courses of study?	Survey/ Semi-longitudinal	Self-reported measure of Type A behavior, and measures of involvement with premedical studies	Premedical students and non-premedical students from 13 private, liberal arts colleges in the Associated Colleges of the Midwest.	Total sample 253 students (118 premed, 73 bio/chem majors, 62 other majors)	47.9% of initial sample responded to questionnaire; Groupings made to resurvey a subset of 336 where 75.3% responded.	Limited generalizability – low response rate, sampled disproportionally from predetermined groups and sampled from small private liberal arts college; Selective attrition
Pascarella et al 1987^30 ^	What are the direct and indirect effects of undergraduate college experience on occupational attainment in medicine?	Survey/ Longitudinal- Qualitative causal modeling	Self-reported measures of family background, secondary school experiences, initial occupational aspirations, and personal characteristics	National sample of premedical students, enrolled in a 4 year undergraduate institution in 1971, who responded to national CIRP survey; followed over 9 years.	Total sample 454 premedical students	Secondary data analysis (n/a)	Selective attrition- difficult to assess effects of aspiration changes with single follow-up; Secondary analysis of existing data- survey not designed around research question and weak operational definitions model variables. (i.e., the 4 college experience variables limited in how they assess the “experience” of the student.)
Sade et al 1984^34^	Does the anecdotally documented “premedical syndrome” exist in reality and what traits comprise is?	Study 1: Survey/Cross-sectional; Study 2: Survey/Cross-sectional	Self-reported ratings of premedical students on 14 different traits, and self-reported major of study	Study 1: Premedical and non-premedical students from 13 colleges in South Carolina; Study 2: Faculty members 13 colleges in South Carolina	Study 1: total sample 498 students; (253 premed, 245 randomly sampled non-premed); Study 2: 403 faculty	Study 1 & 2: Potential participants approached – not reported	Limited generalizability- sampled, small, colleges in one southern state; Data are limited to perceptions and not direct observations; Limited information on items in questionnaire and if it was pretested.
Simmons 2005^37 ^	What are the attitudes of premedical students towards breadth of education?	Study 1: Interviews; Study 2: Focus group interviews with subset of Study 1 participants	Interviews and focus groups focused on student attitudes towards educational breadth	Study 1: Premedical students, junior and senior years, Centerville University	Study 1:15 students; Study 2: 7 students	Study 1:23 of 1100 (.02%) students responded to initial solicitation; 16 (69%) completed interview and 1 eliminated based on study criteria; Study 2: Not reported.	Limited generalizability- sampled one university, focused on liberal arts education; Self-selection bias due to small sample size drawn from large web recruitment effort; Selection bias due to purposeful sampling technique; Selection bias towards students who remain in the premed program because sample of upper classmen; Skewed male/female ratio.
Staley and Hood 1977^22^	What are the causes of higher attrition rates for women in premedical pro-grams?	Longitudinal survey	Self-reported measures of student family characteristics, personal and educational background, and past and future medical career plans	Premedical students, in their freshman and sophomore years, University of Iowa	Total sample 188 students (99 female, 89 male)	Initial response rate to survey invitation not reported; Sample population at T1:89% of females (106); 81% of males (98); Sample population at T2: 93% females (99); 91% males (89)	Limited generalizability- sampled one, large Midwestern university; Selective attrition; Sampling times exclude measurement of changes to perceptions over time after sophomore year; Limited information on items in questionnaire and if it was pretested.

By contrast, more recent work examined the ways premedical students are pushed out of medical careers by negative experiences with required coursework. These studies focus on the attrition of under-represented minorities from the premedical track, demonstrating that negative experiences with required chemistry courses – especially for women from under-represented minority groups – drove students out of the premedical track.^27^^-^^29^ Unlike those described in earlier studies, these students remained interested in medicine, but left the premedical track because they believed they could not survive medical school courses. These more recent studies saw attrition from the premedical track, especially on the part of women and under-represented minorities, as the result of structural factors, rather than lack of interest or motivation on the part of the students.

Three separate articles offered a slightly different perspective on student survival during the premedical years. Pascarella and colleagues found that the quality of the undergraduate institution influenced success in gaining admission to medical school.^30^ While most other articles focused on premedical students in general, this article examined how different undergraduate institutions influenced the students’ progress from the premedical to medical stage. Klink et al., documented the importance of a premedical student’s support network for survival through the premedical years: students with better familial support were more likely to believe in their own coping efficacy.^31^ Finally, Chuck surveyed premedical students and found that most were excessively idealistic about the daily work of a physician, when compared with practicing physicians, suggesting that an idealistic view of medicine may contribute to increasing numbers of medical school applications.^32^  With regards to idealism, it is interesting to note that several studies found that many premedical students report a desire to help others as both a reason to pursue medicine,^32^^,^^33^ as well as a reason to leave the premedical track when they come to believe that their desire to help others can no longer be fulfilled by pursuing a career in medicine.^25^^,^^26^^,^^29^

### The premedical personality—stereotypes and the “premedical syndrome”

The remaining eight of the nineteen articles examined the personality traits of premedical students, exploring the concept of a premedical stereotype or “syndrome.” Studies done in the 1970s and early 1980s depicted the personality traits of premedical students negatively. These studies reported that premedical students were less social and more concerned with money and prestige than other students, claiming that both non-premedical and premedical students saw premedical students as overly competitive, excessively grade-conscious overachievers, with narrow academic interests.^33^^,^^34^ This body of research also found that premedical students concentrated primarily in the sciences, rather than diversifying their academic profiles. Manaster and colleagues found this mentality to be more prevalent during the earlier half of the premedical years. Once premedical students applied to medical school, according to this study, they matured and became more self-assured, demonstrating less of these stereotypical qualities.^35^

Studies published in the mid-1980s to 2010 discovered that the premedical stereotype was more a perception than an observed reality. These studies found that while premedical students were competitive and cared a great deal about grades, they were no different in these respects from other students planning for graduate study in the biological or physical sciences.^36^^,^^37^ Furthermore, unlike previous studies that characterized premedical students as narrowly focused, these studies found that premedical students valued enrolling in a broad range of courses – to a greater extent than other biology or chemistry majors – and cared about mastering course content, rather than working exclusively for the grade.^38^^,^^39^ Conrad found that while many premedical students recognized the stereotype of premedical students as cutthroat and competitive, most premedical students did not actually exhibit cutthroat or competitive behavior.^40^ Instead, many participated in cooperative efforts with their classmates. According to Conrad, the premedical stereotype was a construct that premedical students used to understand their failures and successes within the context of an extremely competitive academic environment.

Taken together, these studies demonstrated that premedical students’ behaviors and motivations are more complicated than they appear at first glance. Those who choose to pursue medicine often balance the necessity of maintaining competitive grades in rigorous coursework against a desire to take a wide variety of classes and develop mastery over course content.

### Limitations of existing research

There are several limitations to the research we reviewed. Most striking is the paucity of empirical studies of the premedical students in the US and the dated nature of the research. Of the nineteen studies included in the review, only ten were published after 1990. Many questions about the premedical experience remain unanswered and at most, the existing literature provides a basis for comparison to today’s students.

Additionally, differences in sampling strategies across studies make it particularly difficult to draw general conclusions about premedical students. It is worth noting that the premedical student population is particularly challenging to identify and sample, since anything prior to medical school could be considered “premedical.” In fact, studies used a variety of definitions of a premedical student to identify their samples of interest. Some studies used the Association of American Medical Colleges (AAMC) data on the MCAT to identify their samples.^25^^,^^26^ Others used enrollment in premedical-required courses as indicators.^23^^,^^24^^,^^31^ Those authors with connections to the premedical advising structure used email lists and social networks to recruit participants.^34^^,^^37^^,^^40^ The most common sampling method was self-identification by the student.^22^^,^^27^^-^^30^^,^^32^^,^^33^^,^^35^^,^^36^^,^^38^^,^^39^Each of these methods yielded slightly different samples of premedical students, which, in turn, can influence the conclusions drawn.

Future studies should attempt to sample all types of premedical students, including those who eventually leave the premedical track. Furthermore, if we are to understand how premedical students identify themselves and the factors that influence attrition from the premedical track we must also follow samples of premedical students through their college careers, observing how bright-eyed and eager first-year premedical students are shaped by their college experiences.

## Discussion

While based on sparse information, our review demonstrates that research on premedical students has focused primarily on two questions: why do students leave the premedical track and what are the attributes and personality characteristics of a premedical student? Research suggests that in the 1970s through the 1980s attrition was linked to students’ decreased desire to pursue a medical career. More recent studies found that negative experiences with coursework, and particularly with chemistry classes, led students to leave the premedical track. The premedical stereotype – a largely negative image of premedical students as single-minded, competitive, and obsessed with grades – continues to have currency inside and outside the premedical world, in spite of research that shows that premeds are, in fact, a diverse group of students with a broad range of interests.

The studies reviewed suggest that the premedical experience may be difficult and overly competitive, leading some would-be doctors to seek other careers. According to this research, the premedical experience is shaped by the participation of high-achieving, goal-oriented – and in some instances, overtly competitive-premedical students in challenging curricular requirements, creating an environment that may ultimately dissuade some students from applying to medical school. For students with unrealistic expectations of careers in medicine, participation in such an environment may result in an appropriate decision to change careers. However, it is also possible that some capable and dedicated students who are not ready for the rigorous demands of premedical coursework may be pushed into an inappropriate early decision to abandon their career aspirations. Thus these studies highlight the potential role of the premedical experience in pushing potentially capable doctors out of the premedical track.

## Conclusions

Our critical review of the literature underscores the need for up-to-date, high quality empirical research on the premedical experience. Some of the research reviewed here was published nearly 40 years ago. Over the past four decades medicine has seen dramatic changes: increasing numbers of women and underrepresented minorities entering the profession and dramatic shifts in the financing and organization of healthcare that have altered the requirements for, and the experience of, premedical and medical education.41 Studies of premedical students conducted many years ago likely have little application to current premedical education experiences, particularly for women and underrepresented minorities. Additionally, medical education itself has changed, becoming broader and more interdisciplinary, emphasizing the social determinants of health, doctor-patient communication and bioethics, in addition to anatomy and physiology. One need look no further than the recent changes to the MCAT, with the addition of two new test sections emphasizing the social sciences, to see the changes undergoing the medical education system.^5^ We need updated research to examine how the premedical experience in this new context of medical education is shaping the physicians of tomorrow.

We also need more empirical research in medical education to focus on the premedical years. To our knowledge, our review is the first to critically synthesize information from the empirical literature on premedical students and we found few articles that met our search criteria. Though premedical education is a topic about which much has been written, most of that writing has been in either in the form of opinion pieces or published in national reports of various professional organizations.^42^ Thus, the few articles that met our search criteria is an important and also serendipitous finding of our review, confirming our larger point that we have much to learn about the how the premedical years shape the physician workforce.

Interestingly, our review also identifies a shift in the conceptualization of the premedical student and the premedical experience over time. Earlier studies of premedical students focused on the individuals who choose to pursue premedical studies at the undergraduate level. These studies show premedical students to be overly ambitious, to the point of being perceived as cutthroat, or likely to leave premedical studies because of a growing disaffection with medicine. These studies focused on the individual-the premedical experience is described as a function of individual personality traits or individual aspirations, with little attention paid to the contextual influences on premedical students. More recent literature, however, recognizes the importance of context for the premedical experience. These studies note that the premedical experience is shaped, not just by personal characteristics, but also by formal curricular requirements and strong social norms that influence the identity of an ideal and successful premed student. While we lack the data to explain this shift in research focus, this change may be the result of the increasing influence of medical sociology on studies of medical education, as well as increasing attention to the influences of the hidden curriculum on the part of medical educators.^43^^,^^44^

On a related note, it is interesting to note that research has primarily focused on two topics – attrition and the premedical stereotype–leaving much room for other research questions regarding how the premedical experience influences those who wish to become doctors. One topic that deserves special attention is the hidden curriculum of the premedical years. We need studies designed to understand the overall culture of premedical education and not just the traits of individual premedical students. While there is a significant body of literature demonstrating the existence of the rich subcultures that emerge among both medical students and residents in response to stresses in the environment,^45^^-^^47^ we were unable to find similar studies at the undergraduate level. Therefore, we know little about premedical culture-such as the survival strategies students collectively develop during this period – and the effects of that culture on future physicians. Rather than continue to focus disproportionately on the academic content of the formal premedical curriculum, new research should pay attention to the informal and hidden curricula—the tacit knowledge premedical students learn informally from advisors, parents, and peers—from watching what they say as well as what they do.^16^

In short, our review demonstrates a need for more high-quality and updated research on the premedical years, and in particular research that focuses on the premedical experience. We know more about the personal characteristics of premedical students than we do about the premedical subculture. We know more about the formal curriculum than about the hidden curriculum. Furthermore, we know little about those who are not admitted and still less about those who abandon their premedical aspirations. According to the AAMC, there were 42,742 applicants for approximately 19,000 seats in medical school in 2010.^48^ This group, however, was drawn from an even larger, unknown number of college undergraduates who participated in the undergraduate premedical experience but did not apply to medical school. If we wish to influence the character of future physicians we must pay attention to what happens to students on their way to medical school. We must explore how this period influences students’ ideas about success, relationships, and caring for others.^15^ In order to gain a deeper and more complete understanding of the physicians of the future, researchers must give equal attention to the first, critical steps occurring during the professionalization process: the premedical years.

### Acknowledgments

We thank Whitney Townsend, the coordinator for the Health Sciences Executive Research Services, for her assistance on conducting our database searches. We would like to thank Erica Blom for her research assistance in the initial stages of the search. Ms. Lin is supported in part by a grant from the Office of the Vice President of Research and an NIA training grant to the Population Studies Center at the University of Michigan (T32 AG000221), University of Michigan, Mr. Crawford by the Center for Ethics in Public Life, University of Michigan and Dr. De Vries is supported in part by a grant from the National Library of Medicine (1G13LM008781-01).

### Conflicts of Interest

The authors declare that they have no conflict of interest.

## Supplementary material

Supplementary file 1 Appendix I. Databases searched and search strategies
