# Supplementary material for: The undergraduate premedical experience in the United States: a critical review
Source: Int J Med Educ. 2013 Feb 10;4:26–37. doi: 10.5116/ijme.5103.a8d3 (PMC3742104; doi:10.5116/ijme.5103.a8d3)
Supplement: Supplementary file 1 — Appendix I. Databases searched and search strategies [file ijme-4-26-S1.pdf]

Appendix I

Databases searched and search strategies

ERIC

KW = premed* OR DE = ("premedical students") OR (DE = ("medical education") AND DE = ("undergraduate study"))

JSTOR

premedical

PubMed

(race/ethnicity)

"Education, Premedical"[Mesh] AND ("race"[All Fields] OR "ethnicity"[All Fields] OR "ethnic"[All Fields] OR "racial"[All Fields] OR "diversity"[All Fields] OR "minority"[All Fields] OR "Continental Population Groups"[Mesh] OR "Race Relations"[Mesh] OR "Asian Continental Ancestry Group"[Mesh] OR "European Continental Ancestry Group"[Mesh] OR "Oceanic Ancestry Group"[Mesh] OR "African Continental Ancestry Group"[Mesh] OR "Ethnic Groups"[Mesh])

gender

"Education, Premedical"[Mesh] AND ("Gender"[All Fields] OR "Female"[Majr] OR "Male"[Majr] OR "Gender Identity"[Mesh])

history

"Education, Premedical"[Mesh] AND ("History"[Mesh] OR "history"[Subheading])

Scopus

TITLE-ABS-KEY(premedical AND education)

ISI Web of Science

Topic = (premedical AND education)

PsycInfo

(premed* AND education) OR premedical
